# Clinical presentation and hospitalisation risk of RSV in primary care among children younger than 5 years in Italy in four seasons between 2019 and 2023: a multicentre prospective cohort study

**DOI:** 10.1016/j.lanepe.2026.101652

**Published:** 2026-03-24

**Authors:** Francesco Baglivo, Sara Bracaloni, Enrica Esposito, Federico Tecchio, Beatrice Casini, Mauro Pistello, Donatella Panatto, Andrea Orsi, Elvira Massaro, Maria Chironna, Francesca Centrone, Elena Pariani, Laura Pellegrinelli, Elisabetta Pandolfi, Ileana Croci, the RSVComNet Ita Network, Nicolò Di Gaddo, Michela Scarpaci, Tommaso Cosci, Luigi De Angelis, Melissa Torrisi, Leonardo Bonaldo, Guglielmo Arzilli, Gianluca Paparatto, Virginia Casigliani, Andrea Davide Porretta, Federica Chiovelli, Yasmine Ferchichi, Maria Sidoti, Anna Lisa Capria, Valerio Corsini, Flavia Favilli, Paola Mazzetti, Loredana Costabile, Rossella Cannavò, Micaela Foco, Valentina Grimaldi, Immacolata Labella, Roberta Lanni, Donatella Morano, Paolo Nardini, Fabrizio Piperno, Claudia Pontesilli, Innocenza Rafele, Laura Reali, Teresa Rongai, Michele Valente, Laura Venuti, Piero Luigi Lai, Carola Minet, Carlo Simone Trombetta, Chiara Amoruso, Alessia De Marzo, Miriana Girardi, Lucia Servedio, Lucia Peccarisi, Giovanni Capaldi, Daniela Damiani, Vincenzo Frappampina, Stefania Frau, Nunzio Guglielmi, Giuseppe Ragnatela, Monica Pepe, Cristoforo Vania, Cristina Galli, Sandro Binda, Paola Guidotti, Arianna Passoni, Patrizia Rogari, Luigi Greco, Caterina Rizzo

**Affiliations:** a Department of Translational Research and New Technologies in Medicine and Surgery, University of Pisa, Pisa, Italy; b Department of Health Sciences, University of Genoa, Genoa, Italy; c Hygiene Section, Department of Interdisciplinary Medicine, University of Bari “A. Moro”, Bari, Italy; d Hygiene Unit, Azienda Ospedaliero-Universitaria Consorziale Policlinico of Bari, 70124, Bari, Italy; e Department of biomedical Sciences for Health, University of Milan, Milan, Italy; f Predictive and Preventive Medicine Research Unit, Bambino Gesù Children's Hospital, IRCCS, Rome, Italy

**Keywords:** Respiratory syncytial virus (RSV), Pediatrics, Primary care, Acute respiratory infection (ARI), Hospitalization risk, Prospective cohort study, Epidemiology, Italy, Surveillance, RSV clinical presentation, Public Health

## Abstract

**Background:**

Respiratory syncytial virus (RSV) significantly contributes to pediatric morbidity globally. Severe cases are more common in infants and children with underlying health conditions, but even mild infections can result in long-term respiratory issues. While the RSV disease is well-documented in hospital settings, its clinical presentation and hospitalization risk in primary care, the initial point of healthcare access, remains unclear.

**Methods:**

We conducted a multi-center prospective cohort study across multiple regions in Italy during four seasons (from 2019 to 2020 to 2023–2024), excluding 2020–2021. Children ≤5 years with acute respiratory infection (ARI), meeting the WHO case definition for community-based surveillance, were enrolled through primary care pediatricians. Nasopharyngeal swabs were collected and tested with RT-PCR. Clinical data were collected at baseline for all participants and at follow-up intervals of 14 and 30 days post-enrollment for RSV-positive children.

**Findings:**

Among 1410 enrolled children with ARIs, RSV was laboratory-confirmed in 40.2% (n = 566). The mean duration of illness among RSV-positive children was 15.2 days (SD 8.8 days), with symptoms persisting in 40.9% of cases at day 14 and 15.4% at day 30. Hospitalization occurred in 4.4% (25/566) of RSV-positive cases, with a median hospital stay of 5 days (IQR: 4–7 days). Younger age was identified as the primary predictor of hospitalization (observed hospitalization rate of 16.2% in children under 6 months), with an estimated hospitalization risk of 12.5% at birth (95% CI: 3.4–33.1%). Fever did not differentiate RSV infection from other respiratory pathogens (p = 0.084).

**Interpretation:**

Our study highlights the significant hospitalization risks associated with RSV infections among children presenting in primary care settings, offering detailed age-specific risk estimates crucial for accurate health technology assessments (HTAs) of preventive strategies. The findings strongly support exploring the expansion of RSV preventive interventions beyond the conventional age threshold of 24 months, considering the persisting risk in older children. Furthermore, we emphasize the importance of adopting a case definition without a mandatory fever criterion to enhance the sensitivity of RSV surveillance and thus improve the validity of disease-related estimates.

**Funding:**

RSV ComNet is a collaborative study funded by Sanofi and AstraZeneca. Study design and planning were undertaken in collaboration with Sanofi researchers. All data collection, analyses, interpretation, manuscript drafting, and the decision to submit for publication were performed independently by the authors. No datasets were shared with the funding parties.

**Research in context**

**Evidence before this study**

Using the MeSH terms (“Respiratory Syncytial Viruses”) and (“child, preschool” OR “infant”) and (“Hospitalization”) and (“Prospective Studies”) on Mar 21st, 2025, we searched on PubMed for studies published between Jan 1st, 2015, and Mar 21st, 2025 with no language restrictions. The search yielded 101 studies, the majority of which were retrospective, hospital-based cohorts, and focused on children admitted with severe acute respiratory infections (SARI) who tested positive for Respiratory Syncytial Virus (RSV). By contrast, the RSV Consortium in Europe (RESCEU) reported results from a prospective, community-enrolling nested cohort study with an RSV-confirmed hospitalization rate of 1.8% (95% CI 1.6–2.1%) within the first year of life during 2017–2021. However, emerging evidence indicates that the COVID-19 pandemic has altered RSV epidemiology, with a shift toward older age at infection in the post-pandemic period.

**Added value of this study**

To our knowledge, RSV ComNet ITA is the first prospective multicentre study conducted in a primary care setting, providing estimates on hospitalization risk for laboratory-confirmed RSV infections among children under 5 years.

We enrolled over 1400 children meeting the World Health Organization (WHO) acute respiratory infection (ARI) definition for community-based surveillance of RSV infection and prospectively monitored 566 (40.2%) RSV-positive cases for 30 days. Hospitalization occurred in 4.4% of RSV cases due to worsening clinical conditions, with a median stay of 5 days.

Our findings confirmed younger age as the primary predictor for hospitalization, with an estimated hospitalization risk of 12.5% (95% CI 3.4–33.1%) at birth, decreasing to less than 1% (95% CI 0.0–4.1%) at 54 months of age. Consistent with prior evidence that fever is not always present in RSV infection, our data showed that this symptom, despite its common inclusion in surveillance case definitions (SARI and ILI), did not discriminate RSV from other respiratory pathogens (p = 0.084).

**Implications of all the available evidence**

Refined epidemiological definitions and accurate age-specific hospitalization risk data are crucial for accurately determining the clinical and economic impact of preventive interventions, such as maternal vaccinations and monoclonal antibodies.

Furthermore, our findings support the adoption of the WHO ARI case definition, which does not mandate fever, for consistent RSV surveillance in primary care settings. The adoption of standardized surveillance definitions is needed for assessing vaccine effectiveness and understanding the broader public health impact of preventive measures recently implemented.

## Introduction

Respiratory syncytial virus (RSV) is a ubiquitous and highly contagious respiratory pathogen that affects all age groups, exerting a significant clinical burden, particularly in the pediatric population.^1^ RSV is a leading cause of acute respiratory infections (ARIs), including bronchiolitis and pneumonia, particularly in children under 5 years of age.^2^^,^^3^ Severe RSV infection is also more likely to occur in children with underlying medical conditions, such as congenital heart disease, chronic lung disease, and premature birth.^4^ The global impact of RSV is substantial, with an estimated 3.6 million hospitalizations and about 100,000 deaths in children under 5 years of age each year.^5^ While severe cases predominantly affect infants under one year of age and children with underlying health conditions, RSV infections, even when mild, are linked to long-term respiratory complications such as recurrent wheezing and increased asthma risk.^6^^,^^7^ RSV exhibits a distinct seasonal pattern, with annual epidemics occurring during the colder months in temperate regions, typically peaking between late autumn and early spring.^8^ In Italy, RSV circulation generally spans from October/November to March/April, with peak incidence in January/February, partially overlapping with the influenza season.^9^^,^^10^ The COVID-19 pandemic measures caused considerable disruption to the RSV seasonal cycle, with increasing transmission rates in all population groups and an earlier-than-usual start of the season in the post-pandemic years; moreover, the age range of children susceptible to RSV widened.^11^, ^12^, ^13^ RSV is classified into two main antigenic subtypes, RSV-A and RSV-B, which co-circulate during most seasons.^14^ While RSV-A tends to predominate, some studies have reported alternating trends in subtype prevalence without significant differences in clinical severity.^15^

Following an incubation period of 4–6 days, RSV infection typically presents with influenza-like symptoms, including cough, wheezing, shortness of breath, fever, sore throat, and feeding difficulties. However, the specific clinical burden of RSV in primary care settings is poorly understood, with limited data on symptoms and illness duration, particularly in countries like Italy where ILI-based surveillance predominates.^16^, ^17^, ^18^ In Europe, approximately 14.1% of healthy term infants visit a doctor due to RSV in their first year of life, and 1.8% are hospitalized.^19^ Yet, comprehensive studies, such as the multi-country RSVComNet project, highlighted the need for deeper insights into RSV's clinical burden in primary care. The project started in 2019 and was based on a standardized protocol to measure the clinical and socio-economic disease burden of RSV in young children (aged <5 years) in primary care in several European countries.^20^

Significant advances in immunization strategies have supported efforts to reduce RSV's burden. Palivizumab was the sole monoclonal antibody available for years, targeting high-risk groups of newborns and infants. Recently, nirsevimab (at the end of 2022) and clesrovimab (in September 2025) were approved in Europe by the EMA (European Medicines Agency) for both term and preterm infants.^21^^,^^22^ The introduction of vaccines has further transformed the landscape: RSVpreF, a bivalent RSV prefusion F subunit vaccine, has been approved by the EMA in summer 2023 for maternal administration during the third trimester, providing passive protection to newborns.^23^ Additional vaccine candidates, mRNA, live-attenuated, subunit, and recombinant vector platforms, are in various stages of development.^24^ As the World Health Organization (WHO) and the European Center for Disease Prevention and Control (ECDC) advocate, robust population-based surveillance systems are essential to optimizing RSV immunization strategies and resource allocation.^25^ The Italian arm of the RSVComNet multi-country project explored the seasonality pattern and the clinical and socioeconomic burden of RSV in the pediatric population in various Italian regions during different seasons, starting during the pre-pandemic winter season^26^ and continuing through the post-pandemic seasons 2022–23^27^ and 2023–24.

The present study combines data from four seasons (2019–2024) in Italy to determine the symptoms at the clinical presentation of RSV infection compared to other respiratory viruses and the risk of hospitalization due to RSV disease in children under five years of age accessing primary healthcare.

## Methods

### Study design and setting

We conducted a prospective, multi-center cohort study across various Italian regions (Tuscany, Lazio, Liguria, Lombardy, and Puglia) over four seasons, from 2019 to 2020 to 2023–2024, except for the 2020–2021 season, due to difficulties related to the COVID-19 pandemic. The study was conducted in primary care settings at multiple sites in each region.

In Italy, primary care pediatricians provide general, first-access care for children and adolescents aged 0–14 years. Patients were recruited exclusively through primary care pediatricians. Meetings with representatives of local pediatricians' organizations were conducted in each region to promote enrollment and explain the study's design and goals; participation by pediatricians was voluntary. For each season, enrollment windows were aligned with national respiratory virus surveillance and opened once local ethics approvals were in place.^28^ Table 1 provides details about the participating regions, the number of pediatricians, and the study period for the four different study seasons.

**Table 1:** Study sites, recruitment periods, and participating pediatricians across seasons.

Season	2019–2020	2021–2022	2022–2023	2023–2024
Study period (weeks)	W47–W13^b^	W44–W17^b^	W44–W13^b^	W48–W15^b^
Region				
Puglia	participating	participating	participating	participating
Toscana	–	–	participating	–
Lazio	participating	participating	participating	–
Liguria	–	participating	participating	participating
Lombardia	–	participating	participating	participating
Total population (age ≤5 years)^a^	447,857	933,853	1,050,573	646,639
Participating pediatricians				
n° of pediatricians	13	30	55	24
total assisted children (% over total population)	4400 (0.98%)	10,100 (1.08%)	18,500 (1.76%)	9800 (1.51%)
Availability of online platform	–	–	available	available

a Refers to the resident population in participating sites on the 1st of January, included in the study period. Data is publicly available and retrieved from the ISTAT database https://demo.istat.it/ (accessed 09-03-2025).

b Dates are expressed as ISO-8601 calendar weeks (Monday–Sunday).

### Patient recruitment and case definition

For this study, we used the WHO-ECDC ARI case definition for community-based surveillance of RSV infection,^29^ which is defined as an acute respiratory infection having at least one of the following symptoms: shortness of breath, cough, sore throat, and coryza, and a clinician's judgment that the illness is due to an infection. Primary care pediatricians recruited patients during their ordinary outpatient activity using convenience sampling. Caregivers of eligible children were invited to participate, and after obtaining written informed consent, a baseline (T0) clinical assessment was conducted, along with the collection of a nasopharyngeal swab.

### Inclusion and exclusion criteria

The following are the inclusion and exclusion criteria for participants in the study:

#### Inclusion criteria

Participants are eligible if they present with acute respiratory infection (ARI) as defined in the study case definition, are aged 0–5 years, and the clinician judges that the condition is attributable to an infectious cause. In addition, written informed consent must be obtained from the child's parent(s) or legal guardian(s).

#### Exclusion criteria

Participants are excluded if they are older than 5 years or if the pediatrician's clinical examination is negative for ARI. They are also excluded if the parent(s) or guardian(s) have insufficient knowledge of Italian to complete the T14 and T30 questionnaires, or are unable to complete the T14 and T30 questionnaires due to intellectual limitations or specific personal or family circumstances (as determined by the physician, for example, a recent bereavement). Participants are further excluded if informed consent is not provided or if the child has already had an RSV-positive test during the current season.

### Laboratory testing

Pediatricians were instructed to provide nasopharyngeal swab tests to the recruited patients and to collect information using a specific questionnaire on the day of swabbing (Day 0).

Swabs were collected during regular outpatient visits. Subsequently, samples were sent to a regional reference laboratory in each region, where they were tested using multiplex RT-qPCR (Allplex Respiratory Full Panel Assay, Seegene, South Korea) throughout all seasons. Swabs were classified as negative when no genetic material from the targeted pathogens was detected, while co-infection was classified as a multiplex PCR positive for ≥1 non-RSV pathogen. The test results were communicated to the primary care pediatricians as soon as they became available.

### Baseline (T0) and follow-up questionnaires (T14-T30)

The Day 0 questionnaire (T0) included information about patient demographics, date of onset of symptoms, presenting symptoms, and some relevant medical history of the child. Children with a laboratory-confirmed diagnosis of RSV were followed up by the researchers through telephone interviews with their parents at 14 and 30 days after the swabbing (T14 and T30). T0 symptoms were assessed by medical doctors specialized in child care during the enrollment visit (for the instances of collection for each season, please refer to Supplementary Table S1). The follow-up questionnaires varied slightly across different seasons; in particular, during the first season, the T30 questionnaire focused exclusively on quality of life. In the following seasons, the same questions were asked after 14 and 30 days, regarding the persistence of symptoms, the type of drug therapy used, healthcare utilization, and the socioeconomic impact (refer to Supplementary Table S1 for the list of symptoms). From the 2022–2023 season, all information was stored on a web-based platform created specifically for the study.

### Outcome definitions

Predefined outcome measures were established in accordance with the research protocol for this study. The primary outcome of interest in this paper is hospitalization due to RSV infection, which was defined as hospital admission for at least 24 h due to worsening respiratory symptoms in a child enrolled in the study who tested positive for RSV via PCR laboratory confirmation.

Hospitalization length was defined as the total number of calendar days a child meeting the RSV hospitalization criteria remains admitted, including any transfers to other healthcare facilities.

Illness duration was defined as the total number of calendar days during which an RSV-positive child exhibits symptoms. It was determined by recording the date of symptom onset at the initial assessment (T0) and the date of symptom resolution, as reported by parents during follow-up telephone interviews conducted at T14 and T30.

### Statistical methods

Data from all seasons were reviewed for inconsistencies and merged into a single dataset for analysis. Variables not collected in specific seasons or missing for individual participants were not imputed. We decided to conduct complete case analysis if fewer than 5% of cases were dropped due to missing data in the models described below. Demographic characteristics were summarized as frequencies for categorical variables and as means or medians for continuous variables. Age, sex, prematurity, and chronic respiratory disease status were compared between RSV-positive and RSV-negative children using the Wilcoxon rank-sum test or the chi-square test.

Logistic regression analyses were conducted to identify associations between specific symptoms and predefined groups based on RSV infection and co-infection status; all estimates were contrasted with the reference category of children who met the ARI case definition and had a valid multiplex RT-qPCR result negative for all assayed targets. For each symptom, we analyzed data from children with valid records (i.e., excluding missing and not collected data). The outcome variable was the presence or absence of the symptom, which was binary. Logistic regression models were constructed for each symptom, and odds ratios (ORs) with 95% confidence intervals (CIs) were calculated. Adjustments were made for potential confounding effects of season, region, age, sex, and prematurity. Regarding the logistic regression analyses performed to identify symptoms significantly associated with RSV infection, the forest plot of the odds ratios (OR) for each symptom stratified by pathogen group (regression analyses were not performed for dehydration and fatigue since data for these symptoms were collected only during the 2023–2024 season) was presented. Results were considered clinically significant if the groups “RSV+” and “RSV & other-pathogen”, compared to negative swabs, showed statistically significant differences, while “FLU+” and “other-pathogen” groups' differences with negative swabs were not significant or significant in the opposite direction, or if “RSV+” group difference with negative swabs is in the opposite direction or not significant while all the other groups are statistically significant different from negative swabs. The influenza clinical profile is well known; therefore, the “FLU+” category serves as an internal validity check and a clinically meaningful contrast for interpreting results.

Univariate logistic regression analysis was performed to identify factors associated with hospitalization in children with RSV. A priori relevant variables (age, sex, season, prematurity, and co-infection) were included in multivariable analysis to estimate the hospitalization risk for RSV-positive children. Given the expected class imbalance between hospitalized and non-hospitalized children, a penalized regression model was chosen (elastic net regression). Eleven regressions were computed, with alpha values ranging from 0 (LASSO regression) to 1 (ridge regression), and a 10-fold cross-validation was applied to determine the best lambda at each iteration. The area under the curve (AUC) was calculated for each regression, and the regression with the maximum AUC was selected. Hospitalization probabilities were then imputed using the chosen regression, and 95% CIs were estimated via bootstrapping (n = 300 boots). Calibration of the model and center-specific discrimination are reported in Supplementary S8 and S9.

All statistical analyses were conducted using R statistical software (version 4.2.2). A p-value ≤0.05 was considered statistically significant.

### Ethics approval

The study was conducted in accordance with the principles of the Declaration of Helsinki, and all patients' legal tutors provided written informed consent prior to enrollment. It was first approved in 2019 and has been re-approved every year. The approvals for each year are listed below.

Season 2019–2020: the study was approved by the Medical Ethical Committee of OPBG Medical Center (prot. 1301_OPBG_2019 on 30/09/2019).

Season 2021–2022: the study was approved by the Medical Ethical Committee of OPBG Medical Center (prot. 644_OPBG_2022 on 16/05/2022).

Season 2022–2023: the study was approved by the Ethical Committee “Comitato Etico di Area Vasta Nord Ovest (CEAVNO) per la Sperimentazione clinica” of the Tuscany Region, Italy (prot. 22871_Dini on 25/10/2022).

Season 2023–2024: the study was approved by the Ethical Committee “Comitato Etico di Area Vasta Nord Ovest (CEAVNO) per la Sperimentazione clinica” of the Tuscany Region, Italy (prot. 22871_Dini on 05/10/2023).

### Role of the funding source

RSV ComNet is a collaborative study funded by Sanofi and AstraZeneca. Study design and planning were undertaken in collaboration with Sanofi researchers. All data collection, analyses, interpretation, manuscript drafting, and the decision to submit for publication were performed independently by the authors. No datasets were shared with the funding parties.

## Results

### Study population

During the study period from season 2019–20 to season 2023–24, except the 2020–2021 season, due to the COVID-19 pandemic, as defined in Table 1, 1410 children were enrolled, and 569 (40.3%) tested positive for RSV. RSV positivity ranged from 34% in 2021–22 to 44.7% in 2023–24.

**Fig. 1:**
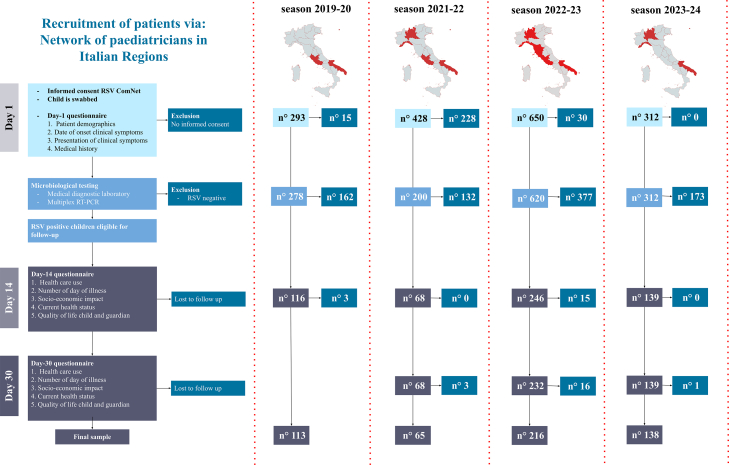
**Flow diagram of participant enrollment and follow-up across study seasons.** The flow diagram depicts participant enrollment, inclusion criteria, and follow-up rates stratified by the four study seasons (2019–2024). Day 1 (T0) represents the baseline assessment performed by primary care paediatricians, while Day 14 (T14) and Day 30 (T30) refer to telephonic follow-ups at 14 and 30 days, respectively. Losses to follow-up are indicated at each stage. Follow-up at T30 for the 2019–2020 season is not reported, as different variables were collected compared to subsequent seasons.

Across all seasons, T0 data were collected from 566 (99.5%) of the RSV-positive children. For 99.8% (n = 842) of RSV-negative children, T0 data were collected. There was a 2.6% (n = 15) loss to the T14 follow-up and a 3.5% (n = 20) additional loss to the T30 follow-up (excluding season 2019–20, where the T30 questionnaire was different). Details on the number of individuals at each stage of study for each season are presented in the flow diagram in Fig. 1, as per STROBE guidelines.

There was a significant difference in age (p = 0.01) between RSV-positive and RSV-negative children. However, no significant differences were observed in sex (p = 0.11), prematurity status (p = 0.31), or the prevalence of chronic respiratory disease (p = 0.21). Table 2 summarizes the demographic and baseline characteristics of enrolled children.

**Table 2:** Baseline characteristics of enrolled children.

	Total	RSV positive	RSV negative
N	1410^a^	569^b^	844^a^
Season			
2019–2020	277 (19.7%)	116 (20.5%)	161 (19.1%)
2021–2022	200 (14.2%)	68 (12.0%)	132 (15.7%)
2022–2023	620 (44.0%)	243 (42.9%)	377 (44.8%)
2023–2024	311 (22.1%)	139 (24.5%)	172 (20.4%)
Region			
Puglia	282 (20.0%)	150 (26.5%)	132 (15.7%)
Toscana	96 (6.8%)	24 (4.2%)	72 (8.5%)
Lazio	274 (19.5%)	122 (21.5%)	152 (18.1%)
Liguria	615 (43.7%)	215 (38.0%)	400 (47.5%)
Lombardia	141 (10.0%)	55 (9.7%)	86 (10.2%)
Demographics			
Median Age in months (IQR)	17 (8–31)	15 (8–29)	18 (9–33)
Age category			
0–5 months	229 (16.3%)	99 (17.5%)	130 (15.4%)
6–11 months	259 (18.4%)	117 (20.7%)	142 (16.9%)
12–23 months	396 (28.1%)	156 (27.6%)	240 (28.5%)
24–59 months	524 (37.2%)	194 (34.3%)	330 (39.2%)
Males	729 (51.8%)	278 (49.1%)	451 (53.6%)
Medical history			
Prematurity (<37 weeks)	125/1404 (8.9%)	43/565 (7.6%)	82/839 (9.8%)
Bronchopulmonary disease	47/1396 (3.4%)	21/564 (3.7%)	26/832 (3.1%)
Symptoms at T0			
Fever	732/1128 (64.9%)	285/447 (63.8%)	447/681 (65.6%)
Cough	1313/1406 (93.4%)	539/564 (95.6%)	774/842 (91.9%)
Coryza	1192/1401 (85.1%)	481/563 (85.4%)	711/838 (84.8%)
Wheezing	365/1131 (32.3%)	196/446 (43.9%)	169/681 (24.8%)
Dyspnea	540/1402 (38.5%)	265/561 (47.2%)	275/841 (32.7%)
Sore throat	404 (28.7%)	135 (23.8%)	269 (31.9%)
Feeding difficulty	407/1130 (36.0%)	171/449 (38.1%)	236/681 (34.6%)
Dehidration	10/311 (3.2%)	5/139 (3.6%)	5/172 (2.9%)
Fatigue	100/311 (32.1%)	46/139 (33.1%)	54/172 (31.4%)
Laboratory results			
RSV	566 (40.2%)	n/a	n/a
A	n/a	293/538 (54.4%)	n/a
B	n/a	239/538 (44.4%)	n/a
A & B	n/a	6/538 (1.1%)	n/a
Other respiratory virus^c^	831 (59.0%)	292 (51.6%)	539 (64.0%)
Influenza	192 (13.6%)	22 (3.9%)	170 (20.2%)
SARS-CoV-2	21 (1.5%)	3 (0.5%)	18 (2.1%)
Other	621 (44.1%)	267 (47.2%)	354 (42.0%)
Negative swab	303 (21.5%)	n/a	303 (36.0%)

All reported as n/N (%), unless indicated otherwise. Abbreviations: n/a: not applicable.

a For two RSV negative children, T0 data were not available and were excluded.

b For three RSV positive children, T0 data were not available and were excluded.

c The total does not sum up since multiple coinfections were identified.

### Clinical presentation of RSV in children

During the study period, the number of symptoms and signs assessed by primary care pediatricians at T0 rose from four to nine. Among these, dyspnea, cough, coryza, and sore throat were collected in all seasons; wheezing, feeding difficulties, and fever in the last three seasons; and finally, dehydration and fatigue were only collected in 2023–24. Among data on collected symptoms, 0.3% on average, were missing, with the highest missing rate being 2.5% for coryza in the 2021–22 season (full details are available in Supplementary Materials).

**Fig. 2:**
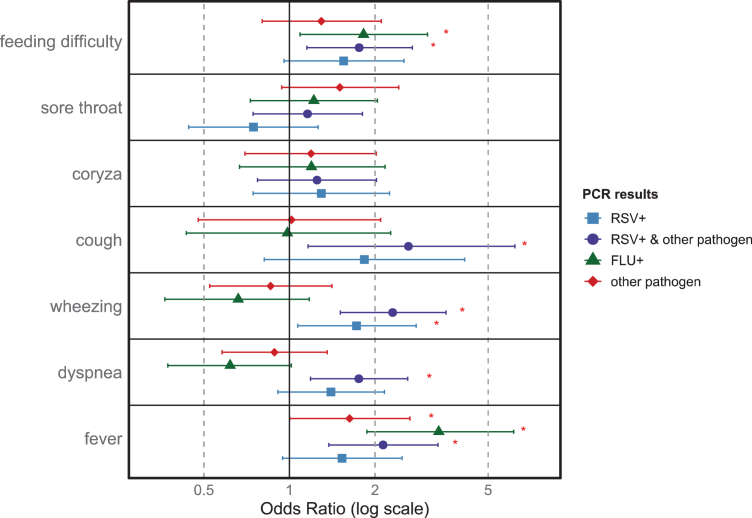
**Association between onset of respiratory symptoms and RSV positivity: logistic regression analysis.** Forest plot of adjusted odds ratios (ORs) with 95% confidence intervals (CIs) for T0 clinical presentation (symptoms at baseline visit) associated with pathogen groups: RSV-only, RSV with co-infections, influenza (A/B), and other respiratory pathogens. ORs were estimated via logistic regression, adjusting for age, sex, season, and geographic region. Each line represents the results of a different regression; complete results are available in Supplementary S3. Reference category: All estimates are compared with the reference category of children who met the ARI case definition and had a valid multiplex RT-qPCR result negative for all assayed targets (“negative swab”). Other pathogens tested: adenovirus; enterovirus; parainfluenza viruses (types 1–4); human metapneumovirus; human bocavirus; rhinovirus; seasonal coronaviruses (HCoV-NL63, HCoV-229E, HCoV-OC43); SARS-CoV-2; *Bordetella pertussis; Bordetella parapertussis; Chlamydophila pneumoniae; Haemophilus influenzae; Legionella pneumophila; Mycoplasma pneumoniae; Streptococcus pneumoniae*. Abbreviations: RSV: respiratory syncytial virus; FLU: influenza virus; OR: odds ratio; CI: confidence intervals.

Cough was the most common symptom among RSV-positive children, reported in 93.4% of cases, followed by coryza (85.1%) and fever (64.9%) (Fig. 2). The full results, including RSV-negative cases, are available in Table 2.

As for the regression analysis, following the criterion described in Methods, significant associations between symptoms at T0 and PCR results were found for:

- Wheezing: significantly associated with both “RSV+” (OR = 1.72, 95% CI: 1.07–2.79; p = 0.03) and “RSV+ & other pathogen” (OR = 2.30, 95% CI: 1.51–3.56; p < 0.0001). In contrast, “FLU+” and “other pathogen” groups did not show a statistically significant difference compared to negative swabs (p = 0.16 and p = 0.54, respectively).
- Fever: significantly associated with “RSV & other pathogen” (OR = 2.13, 95% CI: 1.37–3.33; p = 0.01), “FLU+” (OR = 3.35, 95% CI: 1.87–6.15; p < 0.0001), and “other pathogen” (OR = 1.63, 95% CI: 1.00–2.65; p = 0.05). The “RSV+” group showed no statistically significant difference from negative swabs (OR = 1.53, 95% CI: 0.95–2.49; p = 0.08).

No significant results were observed for the remaining symptoms according to the abovementioned criterion; complete results are available in the Supplementary Materials.

### Illness duration

The mean duration of illness among the RSV-positive children was 15.2 days (SD 8.8; information was available for 96.3% of children, 545/566). There were no statistically significant differences in terms of age, sex, season, or region.

At the T14 interview, 223/545 (40.9%) participants still had symptoms, while at T30, only 84/545 (15.4%). At T14, cough was the most frequently reported symptom, present in 27.1% of cases, followed by coryza at 20.8%. At T30, cough remained the predominant symptom (15.7%), with coryza observed in 10.1% of cases. All other symptoms dropped below 5% at T14 and T30; full details are available in the Supplementary Materials.

### RSV hospitalization risk in children

Among RSV-positive children already seeking primary care attention, 25/566 (4.4%) required hospitalization. The median length of hospitalization was 5 days (IQR: 4–7; range: 2–10 days). The median age of hospitalized children was 4 months (IQR: 2–13; range: 1–57 months). Hospitalization rates varied by season and region; full results are reported in Table 3.

**Table 3:** Characteristics of RSV-hospitalized children and univariate regression results.

	RSV hospitalized	Univariate estimate (std.error)	p-value
n	25/566 (4.4%)		
Season			
2019–2020	7/116 (6.0%)	ref	
2021–2022	5/68 (7.3%)	0.21 (0.61)	0.727
2022–2023	7/243 (2.9%)	−0.77 (0.55)	0.158
2023–2024	6/139 (4.3%)	−0.35 (0.57)	0.536
Region			
Puglia	11/150 (7.3%)	0.62 (0.55)	0.266
Toscana	0/24 (0.0%)	−14.4 (808.00)	0.986
Lazio	5/122 (4.1%)	ref	
Liguria	7/215 (3.2%)	−0.24 (0.60)	0.689
Lombardia	2/55 (3.6%)	−0.12 (0.85)	0.884
Demographics			
Median Age in months (IQR)	4 (2–13)	−0.07 (0.02)	0.002
Age category			
0–5 months	16/99 (16.2%)		
6–11 months	2/117 (1.7%)		
12–23 months	3/156 (1.9%)		
24–59 months	4/194 (2.1%)		
Males	13/25 (52%)	0.12 (0.41)	0.768
Medical history			
Prematurity (<37 weeks)	2/25 (8.0%)	1.06 (0.75)	0.940
Bronchopulmonary disease	0/25 (0.0%)	–	–
Other respiratory virus			
Influenza	0/25 (0.0%)	–	–
SARS-CoV-2	0/25 (0.0%)	–	–
Other	9/25 (36.0%)	−0.67 (0.42)	0.116

Hospitalized children and hospitalization length were assessed through T14 and T30 follow-up questionnaires.

Univariable analyses are shown only for descriptive purposes and were not used to build the multivariable model for hospitalization risk.

The univariate binomial logistic regression performed with RSV hospitalization as the outcome identified younger age as the only significant predictor (p = 0.002). No association was found with season, region, sex, prematurity, or co-infections. The full results are reported in Table 3.

**Fig. 3:**
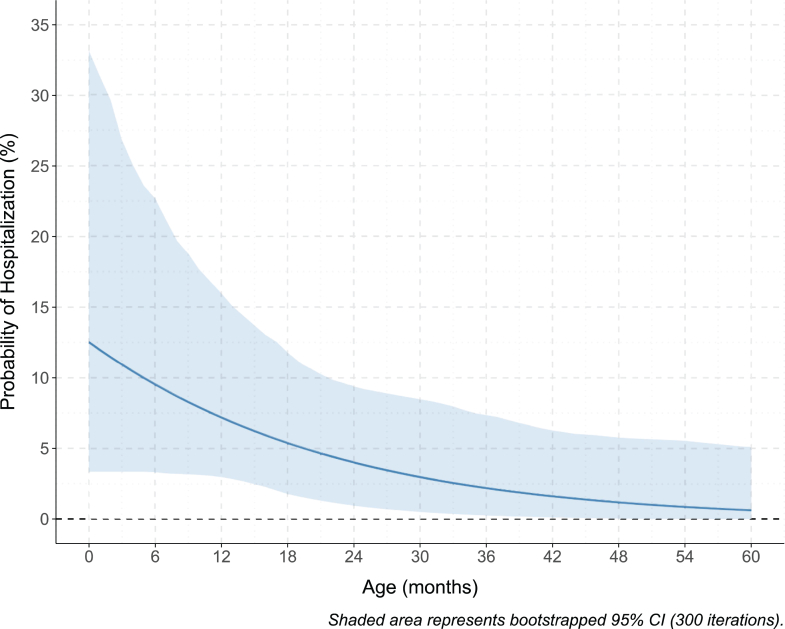
**Predicted probability of hospitalization in children with primary care visits for laboratory-confirmed RSV: Elastic Net regression analysis.** Abbreviations: CI: confidence intervals. Graphic representing a hospitalization probability model in RSV-positive children with outpatient visits, adjusted for sex, prematurity, and season. The model was obtained through Elastic Net regression analysis. The 95% CI is highlighted along the curve. Reference category: children with positive swabs.

However, we a priori included age, sex, prematurity, co-infection, and season in the multivariable analysis due to clinical relevance as described in the methods. The elastic net logistic regression confirmed the role of age as a predictor; complete results are available in the Supplementary Materials. The model was then used to estimate the hospitalization risk of RSV-positive children at different ages, as shown in Fig. 3. The predicted hospitalization risk is 12.5% (95% CI: 3.4–33.1%) at birth, and it becomes lower than 1% (95% CI: 0.0–5.8%) after 48 months of age. Other predicted hospitalization risks are available in the Supplementary Materials.

## Discussion

This multicentric, prospective cohort study conducted over four seasons (2019–2024, excluding the pandemic season) provides detailed epidemiological data on the clinical presentation and the hospitalization risk associated with RSV infection among Italian children under five years presenting to primary care. The study design, encompassing multiple RSV seasons and geographically diverse primary care settings in Italy, strengthens the generalizability of the results to the Italian context. By capturing data directly from primary care, the study addresses a critical gap in understanding the full spectrum of RSV disease, including milder cases that do not typically require hospitalization.^30^ The overall design and burden data from 2019 to 2023 have been discussed by Hak et al.^31^; however, we focus on the case definition for future RSV-specific surveillance in Italy and on assessing hospitalization risk following RSV infection in the Italian context.

This study was conducted before the introduction of the immunization of healthy infants with monoclonal antibodies against RSV in Italy, implemented during the 2024–2025 season.^31^ Future further developments in Italy may include a vaccination campaign targeting children, pregnant women, and adults planned for the 2025–2026 season, with two regions (Sicily and Molise) having already initiated independent vaccination programs.^32^ However, no official recommendations from the Ministry of Health have yet been published on this matter. The findings could therefore represent a baseline for future comparative studies.

RSV was identified as the etiological agent in approximately 40% of ARIs with a mean length of illness of 15.2 days, confirming its significant role as a pathogen in young children under 5 years. This proportion aligns with previous European findings, reinforcing RSV as one of the primary causes of ARI in this age group.^32^^,^^33^ The high infection rate observed in children under 2 years of age underscores the critical need for targeted preventive measures during this vulnerable period, considering nearly all children are infected with RSV by their second birthday.^1^

Notably, approximately 59% of children tested positive for other respiratory pathogens, and approximately 52% of RSV cases presented a co-infection, highlighting the substantial overlap of clinical presentations among respiratory viruses in primary care. The prevalence of co-infections emphasizes the importance of comprehensive diagnostic testing to guide appropriate management decisions and antimicrobial stewardship, particularly in the context of overlapping symptoms and potential for secondary bacterial infections.^34^

Regarding clinical presentation, wheezing emerged as a key symptom significantly associated with RSV infection, observed in 43.9% of RSV-positive children and only in 24.8% of the negative ones. This finding is consistent with international literature, where wheezing is identified as a characteristic clinical feature of RSV, particularly associated with bronchiolitis.^32^

Importantly, despite being commonly reported (64.9% of RSV-positive cases), fever was not significantly associated with RSV infection alone, according to our results. Fever appeared mainly in cases with co-infections, aligning with previous literature suggesting that isolated RSV infections do not typically present with fever as a distinguishing symptom.^32^

Consequently, relying on influenza-like illness (ILI) or SARI definitions in surveillance systems or for estimating hospitalization risk may lead to inaccuracies, as these definitions necessitate the presence of fever.^35^^,^^36^

The hospitalization rate observed in our cohort of RSV-positive children accessing primary care was 4.4%, lower than rates reported in hospital-based studies but consistent with other community-based studies.^37^ This underscores the importance of capturing data from primary care settings to accurately estimate the overall severity of RSV, including milder cases managed in the community.

The median age of hospitalized children in our cohort was 4 months, reflecting existing evidence that younger infants are disproportionately affected by severe RSV disease.^38^ Nevertheless, hospitalizations were observed across all age groups up to 57 months during the study period, indicating that the risk, although reduced, may persist beyond infancy.

Given the prospective nature of our study, we were able to estimate the hospitalization risk due to RSV infection using a multivariable elastic net regression model, confirming age as a significant predictor. Predicted RSV hospitalization risk was highest (approximately 10%) among children younger than six months, decreasing progressively with age, though remaining clinically relevant even in older children. This finding is consistent with previous international reports highlighting that infants younger than six months bear the highest risk of severe RSV disease.^33^ The model demonstrated good discriminatory performance (AUC = 0.75), reinforcing the reliability of the hospitalization risk estimates.

The strengths of this study lie primarily in its prospective multicenter design, standardized data collection across multiple Italian regions, and laboratory confirmation through multiplex RT-PCR, ensuring reliable pathogen identification. Nevertheless, our study has several limitations that should be acknowledged. Firstly, one limitation of the study is the risk of selection bias associated with convenience sampling, which may affect the representativeness of the cohort and the generalizability of the findings. However, in order to minimize this risk, paediatricians were instructed to approach eligible patients during clinic hours and apply a uniform case definition. Another limitation is the variation in recruiting sites across different years, as well as an error in consent collection during the 2021–2022 season, which we reported in Fig. 1 for transparency. Secondly, the absolute number of hospitalized children was limited (n = 25), as expected in a primary care-based study, which may affect the precision of the hospitalization risk estimates and did not allow a reliable multilevel assessment of between-center heterogeneity Furthermore, the rate of hospitalization may be overestimated because the sample population consists of children already accessing primary care. However, the hospitalization risk might also be underestimated due to caregivers bypassing primary care and referring directly to the emergency department. Another limitation of this study is the possibility that the disruption of the RSV seasonal cycle associated with restrictive measures during the COVID-19 pandemic may have influenced the risk of hospitalization due to immune debt.^11^, ^12^, ^13^ However, while epidemiology in several temperate settings may be returning to pre-pandemic patterns, preliminary data from Italy suggest a lingering immune debt in the 2023–24 season.^39^ Thirdly, the number of symptoms assessed by paediatricians at T0 and via phone calls at T14 and T30 follow-up (see Supplementary Table S1) increased during different seasons of the study, with the risk of introducing some degree of measurement heterogeneity. Fourthly, RSV-negative children were not prospectively followed up at 14 and 30 days, restricting the ability to compare the short-term clinical outcomes between RSV-positive and RSV-negative groups. Finally, the model developed was not subject to external validation. Further research should address these limitations to refine hospitalization risk predictions and better understand the long-term sequelae of RSV infection in children under five years of age.

### Conclusions

This prospective cohort study advances our understanding of RSV epidemiology and highlights the crucial role of such studies for evaluating the impact of RSV infection in infants and toddlers, thereby reinforcing the value of collecting primary care surveillance data to inform cost-effectiveness evaluations of preventive strategies.^12^^,^^40^^,^^41^ RSV hospitalization rates following a confirmed RSV infection were substantial, with an approximate risk of 12.5% at birth, progressively decreasing but still considerable beyond 24 months during the study period. Consequently, extending the possibility to vaccinate with new vaccine products that will be available for toddlers and for the older pediatric population should be considered and evaluated through Health Technology Assessment (HTA) studies for achieving a comprehensive reduction of RSV morbidity. By understanding the intricate interplay between RSV infection, clinical presentation, and hospitalization risk, we can strive toward evidence-based policies and targeted interventions that ultimately mitigate the impact of RSV on pediatric health.

Furthermore, our findings underscore that the current definition of ILI employed in RSV surveillance may lack sensitivity, potentially leading to a significant underestimation of the disease burden, as suggested by the WHO. A broader case definition encompassing at least one respiratory symptom without the strict requirement of fever demonstrated improved sensitivity and specificity for detecting RSV infections.^36^ Thus, our results strongly support adopting a more inclusive case definition, explicitly tailored to RSV, to enhance epidemiological accuracy and improve the robustness of RSV surveillance data.

We were able to leverage a robust multi-centric prospective cohort study design that spanned four RSV seasons, ensuring comprehensive and representative data collection. By rigorously applying standardized inclusion criteria, meticulous symptom recording, and multiplex RT-PCR confirmation, we minimized biases and enhanced the reliability of our findings. The study's prospective nature also allowed us to monitor disease progression and assess the incidence of complications, providing valuable insights into the natural history of RSV infection in children under five years old. Moreover, the study benefited from its primary care setting, which enabled the capture of a broad spectrum of disease severity, ranging from mild outpatient cases to those requiring hospitalization, providing a more comprehensive picture of the overall RSV epidemiology.

In the context of pediatric RSV surveillance, refined epidemiological definitions and accurate age-specific hospitalization risk data are crucial for accurately determining the clinical and economic impact of preventive interventions, such as maternal vaccinations and monoclonal antibodies. In conclusion, enhanced surveillance accuracy, paired with detailed stratification by age groups, will facilitate better resource allocation and strengthen public health decision-making.

## Contributors

FB and CR conceived the paper. FB, EE, SB, and FT performed the literature search and drafted the manuscript. FB and FT performed the data extraction, preprocessing, statistical analysis, and visualization. All the authors provided expert insights and contributed to the manuscript revision. CR review the final version of the paper. All the authors approved the final manuscript.

## Data sharing statement

After termination of the ComNet RSV project, anonymized data will be available upon reasonable request. Inquiries can be sent to the corresponding author.

## Declaration of AI and AI-assisted technologies in the writing process

During the preparation of this work, the authors used ChatGPT and Grammarly for English grammar checks. After using this tool/service, the authors reviewed and edited the content as needed and take full responsibility for the content of the publication.

## Editor note

The Lancet Group takes a neutral position with respect to territorial claims in published maps and institutional affiliations.

## Declaration of interests

FB received an educational grant from AstraZeneca and travel reimbursement from Moderna, MSD, and GSK. AO's affiliated institution received grants for conducting clinical trials for GSK, Sanofi, Moderna, Pfizer, MSD, and Sequirus. AO himself received payment for lectures from GSK, Sanofi, MSD, Moderna, Seqirus, Pfizer, AstraZeneca, support for attending meetings and/or travel from GSK, Sanofi, Moderna, Pfizer, MSD, Seqirus, Novavax, Astrazeneca, and participated on an Advisory Board for GSK, Sanofi, Moderna, Pfizer, MSD, Seqirus, Novavax. CR received payment or honoraria for lectures, presentations, speakers bureaus, manuscript writing, or educational events from, and participated in Advisory Board and Expert scientific discussion for Seqirus, MSD, GSK, Sanofi, AstraZeneca, Moderna, Pfizer, Sequirus, Valneva, and Bavarian Nordic.

## Appendix A Supplementary data

Supplementary Tables and Figures

Translated Abstract

Collab Authors
