# Supplementary material for: Clinical presentation and hospitalisation risk of RSV in primary care among children younger than 5 years in Italy in four seasons between 2019 and 2023: a multicentre prospective cohort study
Source: Lancet Reg Health Eur. 2026 Mar 24;65:101652. doi: 10.1016/j.lanepe.2026.101652 (PMC13049889; doi:10.1016/j.lanepe.2026.101652)
Supplement: Supplementary Tables and Figures [file mmc1.docx]

**Supplementary 1. Symptoms assessed by pediatricians at T0, and by telephone interview at T14, and T30 across the considered seasons**

| **Season** | **2019-2020** | **2021-2022** | **2022-2023** | **2023-2024** |
| --- | --- | --- | --- | --- |
| **Symptom T0** |  |  |  |  |
| Fever | nc | collected (1·5%) | collected (0·0%) | collected (0·0%) |
| Dyspnea | collected (2·2%) | collected (0·0%) | collected (0·0%) | collected (0·0%) |
| Wheezing | nc | collected (0·0%) | collected (0·0%) | collected (0·0%) |
| Cough | collected (0·0%) | collected (1·0%) | collected (0·0%) | collected (0·0%) |
| Coryza | collected (0·7%) | collected (2·5%) | collected (0·0%) | collected (0·0%) |
| Sore throat | collected (0·0%) | collected (0·0%) | collected (0·0%) | collected (0·0%) |
| Feeding difficulty | nc | collected (0·5%) | collected (0·0%) | collected (0·0%) |
| Dehydration | nc | nc | nc | collected (0·0%) |
| Fatigue | nc | nc | nc | collected (0·0%) |
| **Symptom T14** |  |  |  |  |
| Fever | nc | collected (5·9%) | collected (6·9%) | collected (0·0%) |
| Dyspnea | collected (3·6%) | collected (5·9%) | collected (6·9%) | collected (0·0%) |
| Wheezing | nc | collected (1·5%) | collected (6·9%) | collected (0·0%) |
| Cough | collected (3·6%) | collected (1·5%) | collected (6·9%) | collected (0·0%) |
| Coryza | collected (3·6%) | collected (1·5%) | collected (6·9%) | collected (0·0%) |
| Sore throat | nc | collected (1·5%) | collected (6·9%) | collected (0·0%) |
| Feeding difficulty | nc | collected (5·9%) | collected (6·9%) | collected (0·0%) |
| Dehydration | nc | nc | nc | collected (0·0%) |
| Fatigue | nc | nc | nc | collected (0·0%) |
| **Symptom T30** |  |  |  |  |
| Fever | nc | collected (1·5%) | collected (1·5%) | collected (0·0%) |
| Dyspnea | nc | collected (1·5%) | collected (1·5%) | collected (0·0%) |
| Wheezing | nc | collected (1·5%) | collected (1·5%) | collected (0·0%) |
| Cough | nc | collected (1·5%) | collected (1·5%) | collected (0·0%) |
| Coryza | nc | collected (1·5%) | collected (1·5%) | collected (0·0%) |
| Sore throat | nc | collected (1·5%) | collected (1·5%) | collected (0·0%) |
| Feeding difficulty | nc | collected (1·5%) | collected (1·5%) | collected (0·0%) |
| Dehydration | nc | nc | nc | collected (0·0%) |
| Fatigue | nc | nc | nc | collected (0·0%) |

Abbreviations: nc: not collected

In season 2019-2020 at T30, symptoms were not collected.

All reported as: collected/nc (missing %).

**Supplementary 2. Complete respiratory pathogen positivity rates by RSV status**

|  | **Total** | **RSV positive** | **RSV negative** |
| --- | --- | --- | --- |
| N | 1410^a^ | 566^b^ | 844^a^ |
| **Laboratory results** |  |  |  |
| RSV | 566 (40·2%) | n/a | n/a |
| A | n/a | 293/538 (54·4%) | n/a |
| B | n/a | 239/538 (44·4%) | n/a |
| A & B | n/a | 6/538 (1·1%) | n/a |
| Other respiratory virus^c^ | 831 (59·0%) | 292 (51·6%) | 539 (64·0%) |
| Influenza | 192 (13·6%) | 22 (3·9%) | 170 (20·2%) |
| SARS-CoV-2 | 21 (1·5%) | 3 (0·5%) | 18 (2·1%) |
| Rhinovirus | 427 (30·2%) | 138 (24·3%) | 289 (34·2%) |
| Enterovirus | 66 (4·6%) | 30 (5·3%) | 36 (4·2%) |
| Adenovirus | 115 (8·1%) | 29 (5·1%) | 86 (10·2%) |
| Bocavirus | 93 (6·5%) | 27 (4·8%) | 66 (7·8%) |
| Coronavirus (OC43, HKU1, 229E, NL63) | 105 (7·4%) | 33 (5·8%) | 72 (8·5%) |
| Parainfluenza virus | 31 (2·1%) | 3 (0.5%) | 28 (3·3%) |
| Metapneumovirus | 162 (11·4%) | 13 (2·3%) | 149 (17·7%) |
| *Haemophilus influenzae* | 201 (14·3%) | 87 (15·3%) | 114 (13·5%) |
| *Streptococcus pneumoniae* | 154 (11·9%) | 64 (11·3%) | 90 (10·7%) |
| Other bacterial^d^ | 5 (0·4%) | 3 (0·5%) | 2 (0·2%) |
| Negative swab | 303 (21·5%) | n/a | 303 (36·0%) |

^a^ for two RSV negative children, T0 data were not available and were excluded

^b^ for three RSV positive children, T0 data were not available and were excluded.

^c^ the total does not sum up since multiple coinfections were identified.

^d^ “Other bacterial” includes: *Chlamydophila pneumoniae, Mycoplasma pneumoniae, Legionella pneumophila, Bordetella parapertussis,* and *Bordetella pertussis.*

**Supplementary 3. Regression results of Clinical Presentation symptoms of RSV in children**

| **Symptom** | **n° with symptom/**  **n° in PCR result group(%)** | **estimated OR (CI 95%)** | **p-value** |
| --- | --- | --- | --- |
| PCR result |  |  |  |
| **Fever** |  |  |  |
| RSV + | 119 / 214 (55·6%) | 1·5318 (0·9455 - 2·4903) | 0·084 |
| RSV + & other pathogen | 166 / 233 (71·2%) | 2·1338 (1·3758 - 3·3304) | 0·0008* |
| FLU + | 110 / 134 (82·1%) | 3·3514 (1·8729 - 6·1533) | 0·0001* |
| other pathogen ^a^ | 165 / 264 (62·5%) | 1·6277 (1·0021 - 2·6523) | 0·0495* |
| negative swab | 172 / 283 (60·8%) | ref. |  |
| **Dyspnea** |  |  |  |
| RSV+ | 127 / 272 (46·7%) | 1·3992 (0·9101 - 2·1583) | 0·1269 |
| RSV + & other pathogen | 138 / 289 (47·8%) | 1·7536 (1·1864 - 2·6041) | 0·0051* |
| FLU+ | 44 / 170 (25·9%) | 0·6181 (0·3731 - 1·0156) | 0·0592 |
| other pathogen ^a^ | 137 / 368 (37·2%) | 0·8855 (0·5791 - 1·3578) | 0·5755 |
| negative swab | 94 / 303 (31·0%) | ref. |  |
| **Wheezing** |  |  |  |
| RSV+ | 92 / 217 (42·4%) | 1·7218 (1·0689 - 2·7916) | 0·0263* |
| RSV + & other pathogen | 104 / 233 (44·6%) | 2·3067 (1·5095 - 3·5560) | 0·0001* |
| FLU+ | 26 / 134 (19·4%) | 0·6601 (0·3644 - 1·1741) | 0·1628 |
| other pathogen ^a^ | 69 / 264 (26·1%) | 0·8583 (0·5242 - 1·4107) | 0·5446 |
| negative swab | 74 / 283 (26·1%) | ref. |  |
| **Cough** |  |  |  |
| RSV+ | 257 / 272 (94·5%) | 1·8342 (0·8154 - 4·1349) | 0·1406 |
| RSV+ & other pathogen | 282 / 292 (96·6%) | 2·6216 (1·1629 - 6·2147) | 0·0227* |
| FLU+ | 157 / 170 (92·4%) | 0·9837 (0·4332 - 2·2715) | 0·9688 |
| other pathogen ^a^ | 337 / 369 (91·3%) | 1·0166 (0·4769 - 2·0920) | 0·9651 |
| negative swab | 280 / 303 (92·4%) | ref. |  |
| **Coryza** |  |  |  |
| RSV+ | 234 / 271 (86·3%) | 1·2938 (0·7445 - 2·2488) | 0·3601 |
| RSV+ & other pathogen | 247 / 292 (84·6%) | 1·2503 (0·7720 - 2·0239) | 0·3625 |
| FLU+ | 143 / 169 (84·6%) | 1·1948 (0·6672 - 2·1707) | 0·5530 |
| other pathogen ^a^ | 316 / 368 (85·9%) | 1·1906 (0·6966 - 2·0214) | 0·5201 |
| negative swab | 252 / 301 (83·7%) |  |  |
| **Sore throat** |  |  |  |
| RSV+ | 53 / 274 (19.3%) | 0·7466 (0·4420 - 1·2609) | 0·2739 |
| RSV+ & other pathogen | 82 / 292 (28.1%) | 1·1576 (0·7450 - 1·8067) | 0·5166 |
| FLU+ | 57 / 170 (33.5%) | 1·2185 (0·7280 - 2·0407) | 0·4517 |
| other pathogen ^a^ | 125 / 369 (33.9%) | 1·5035 (0·9391 - 2·4253) | 0·0916 |
| negative swab | 87 / 303 (28.7%) | ref. |  |
| **Feeding difficulties** |  |  |  |
| RSV+ | 80 / 216 (37·0%) | 1·5513 (0·9564 - 2·5297) | 0·0765 |
| RSV+ & other pathogen | 91 / 233 (39·1%) | 1·7585 (1·1506 - 2·7057) | 0·0096* |
| FLU+ | 57 / 134 (42·5%) | 1·8233 (1·0892 - 3·0603) | 0·0225* |
| other pathogen ^a^ | 92 / 264 (34·8%) | 1·2946 (0·8012 - 2·1039) | 0·2939 |
| negative swab | 87 / 283 (30·7%) | ref. |  |

Abbreviations: RSV: respiratory syncytial virus; FLU: influenza virus; OR: odd ratio.
Symptom refers to T0 symptoms assessed by pediatricians. OR are estimated in comparison to negative swabs through binomial logistic regression ( symptom present ~ PCR result + season + region + age + sex + prematurity).

^a^ Includes Sars-CoV-2 and other respiratory pathogens

**Supplementary 4. T14 and T30 characteristics of RSV-positive children**

|  |  | **Fever** | **Dyspnea** | **Wheezing** | **Cough** | **Coryza** | **Sore Throat** | **Feeding Difficulties** | **Dehydration** | **Fatigue** |
| --- | --- | --- | --- | --- | --- | --- | --- | --- | --- | --- |
| **T14** |  |  |  |  |  |  |  |  |  |  |
| **Season** | | | | | | | | | | |
|  | 2019-2020 | nc | nc | 11/112 (10·0%) | 23/112 (20·5%) | 19/112 (17·0%) | nc | nc | nc | nc |
|  | 2021-2022 | 0/64 (0·0%) | 1/64 (1·6%) | 1/67 (1·4%) | 14/67 (20·9%) | 10/67(14·9%) | 1/67 (1·5%) | 2/64 (3·1%) | nc | nc |
|  | 2022-2023 | 7/229 (3·0%) | 5/229 (2·1%) | 3/229 (1·3%) | 72/229 (31·4%) | 54/229 (23·6%) | 3/229 (1·3%) | 13/229(5·7%) | nc | nc |
|  | 2023-2024 | 1/139 (0·7%) | 0/139 (0·0%) | 2/139 (1·4%) | 39/139 (28%) | 31/139 (22·3%) | 1/139 (0·7%) | 4/139 (2·9%) | 0/139 (0·0%) | 1/139 (0·7%) |
| **Age category** | | | | | | | | | | |
|  | 0-5 months | 0/74 (0·0%) | 2/74 (2·7%) | 6/95 (6·3%) | 24/95 (25·3%) | 25/95 (26·3%) | 1/74 (1·4%) | 2/74 (2·7%) | 0/15 (0·0%) | 0/15 (0·0%) |
|  | 6-11 months | 5/91 (5·5%) | 2/91 (2·2%) | 4/114 (3·5%) | 40/114 (35%) | 35/114 (30·7%) | 1/91 (1·1%) | 4/91 (4·4%) | 0/25 (0·0%) | 0/25 (0·0%) |
|  | 12-23 months | 2/124 (1·6%) | 2/124 (1·6%) | 2/152 (1·3%) | 42/152 (27·6%) | 29/152 (19%) | 1/124 (0·8%) | 7/124 (5·6%) | 0/37 (0·0%) | 1/37 (2·7%) |
|  | 24-59 months | 1/143 (0·7%) | 0/143 (0·0%) | 5/186 (2·7%) | 42/186 (22·6%) | 25/186 (13·4%) | 2/143 (1·4%) | 6/143 (4·2%) | 6/62 (9·7%) | 0/62 (0·0%) |
|  |  |  |  |  |  |  |  |  |  |  |
|  |  | **Fever** | **Dyspnea** | **Wheezing** | **Cough** | **Coryza** | **Sore Throat** | **Feeding Difficulties** | **Dehydration** | **Fatigue** |
| **T30** |  |  |  |  |  |  |  |  |  |  |
| **Season** | | | | | | | | | | |
|  | 2019-2020 | nc | nc | nc | nc | nc | nc | nc | nc | nc |
|  | 2021-2022 | 0/64 (0·0%) | 2/64 (3·1%) | 1/64 (1·6%) | 11/64 (17·2%) | 9/64 (14·0%) | 1/64 (1·6%) | 5/64 (7·8%) | nc | nc |
|  | 2022-2023 | 1/213 (0·5%) | 1/213 (0·5%) | 2/213 (0·9%) | 25/213 (11·7%) | 17/213 (8·0%) | 0/213 (0·0%) | 2/213 (0·9%) | nc | nc |
|  | 2023-2024 | 5/138 (3·6%) | 1/138 (0·7%) | 1/138 (0·7%) | 29/138 (21·0%) | 16/138 (11·6%) | 1/138 (0·7%) | 1/138 (0·7%) | 1/138 (0·7%) | 1/138 (0·7%) |
| **Age Category** | | | | | | | | | | |
|  | 0-5 months | 0/73 (0·0%) | 0/73 (0·0%) | 0/73 (0·0%) | 11/73 (15%) | 6/73 (8·2%) | 0/73 (0·0%) | 3/73 (4·1%) | 0/15 (0·0%) | 0/15 (0·0%) |
|  | 6-11 months | 2/88 (2·2%) | 1/88 (1·1%) | 2/88 (2·2%) | 10/88 (11·4%) | 10/88 (11·4%) | 0/88 (0·0%) | 1/88 (1·1%) | 0/25 (0·0%) | 0/25 (0·0%) |
|  | 12-23 months | 4/117 (3·4%) | 2/117 (1·7%) | 1/117 (0·9%) | 19/117 (16·2%) | 15/117 (12·8%) | 2/117 (1·7%) | 3/117 (2·6%) | 0/36 (0·0%) | 1/36 (0·0%) |
|  | 24-59 months | 1/137 (0·7%) | 0/137 (0·0%) | 1/137 (0·7%) | 25/137 (18·2%) | 11/137 (8·0%) | 0/137 (0·0%) | 1/137 (0·7%) | 0/62 (0·0%) | 0/62 (0·0%) |

Abbreviations: nc: not collected.

Symptoms (fever, dyspnea, wheezing, cough, coryza, sore throat, feeding difficulties, dehydration, and fatigue) refer to T14 and T30 symptoms collected by the investigators through telephone questionnaires submitted to the parents/legal guardians.

**Supplementary 5. Elastic Net Regression grid search and Hospitalization Risk Prediction Model stratified by age**

| **alpha** | **lambda** | **AUC** |
| --- | --- | --- |
| corresponding to RIDGE regression 0·0 | 0·034594597 | 0·7332608 |
| 0·1 | 0·018896528 | 0·7397412 |
| 0·2 | 0·019887690 | 0·7435490 |
| 0·3 | 0·014551144 | 0·7459150 |
| 0·4 | 0·015833413 | 0·7488725 |
| 0·5 | 0·010516142 | 0·7488725 |
| 0·6 | 0·008763452 | 0·7485767 |
| 0·7 | 0·006236204 | 0·7479852 |
| 0·8 | 0·005988698 | 0·7485028 |
| 0·9 | 0·004850381 | 0·7485028 |
| corresponding to LASSO regression 1·0 | 0·005770727 | 0·7498336 |
| **Final model results** |  |  |
| **Selected model** |  |  |
| alpha | 1·0 |  |
| lambda | 0·05 |  |
| **Model parameters (beta)** |  |  |
| age | -0·05123787 |  |
| sex (male) | · |  |
| prematurity (fullterm) | · |  |
| season (2021-2022) | 0·13889306 |  |
| season (2022-2023) | -0·57331936 |  |
| season (2023-2024) | · |  |
| other pathogens | -0.28749764 |  |
|  | **Predicted Hospitalization Risk** | **95% CI bootstrapped** |
| **Age (months)** |  |  |
| 0 | 12·5% | 3·4 - 33·1 % |
| 3 | 10·9% | 3·4 - 26·8 % |
| 6 | 9·5% | 3·3 - 22·7 % |
| 9 | 8·3% | 3·2 - 18·8 % |
| 12 | 7·2% | 3·0 - 16·0 % |
| 15 | 6·2% | 2·5 - 13·7 % |
| 18 | 5·4% | 1·8 - 11·8 % |
| 21 | 4·7% | 1·3 - 10·3 % |
| 24 | 4·0% | 1·0 - 9·4 % |
| 27 | 3·5% | 0·7 - 8·9 % |
| 30 | 3·0% | 0·5 - 8·5 % |
| 36 | 2·2% | 0·3 - 7·4 % |
| 42 | 1·6% | 0·1 - 6·3 % |
| 48 | 1·2% | 0·0 - 5·8 % |
| 54 | 0·9% | 0·0 - 5·5 % |
| 60 | 0·7% | 0·0 - 5·1 % |
|  |  |  |

Abbreviation: AUC: Area Under Curve

**Figure S5. ROC curve of the Elastic Net Regression Hospitalization Risk Prediction Model**


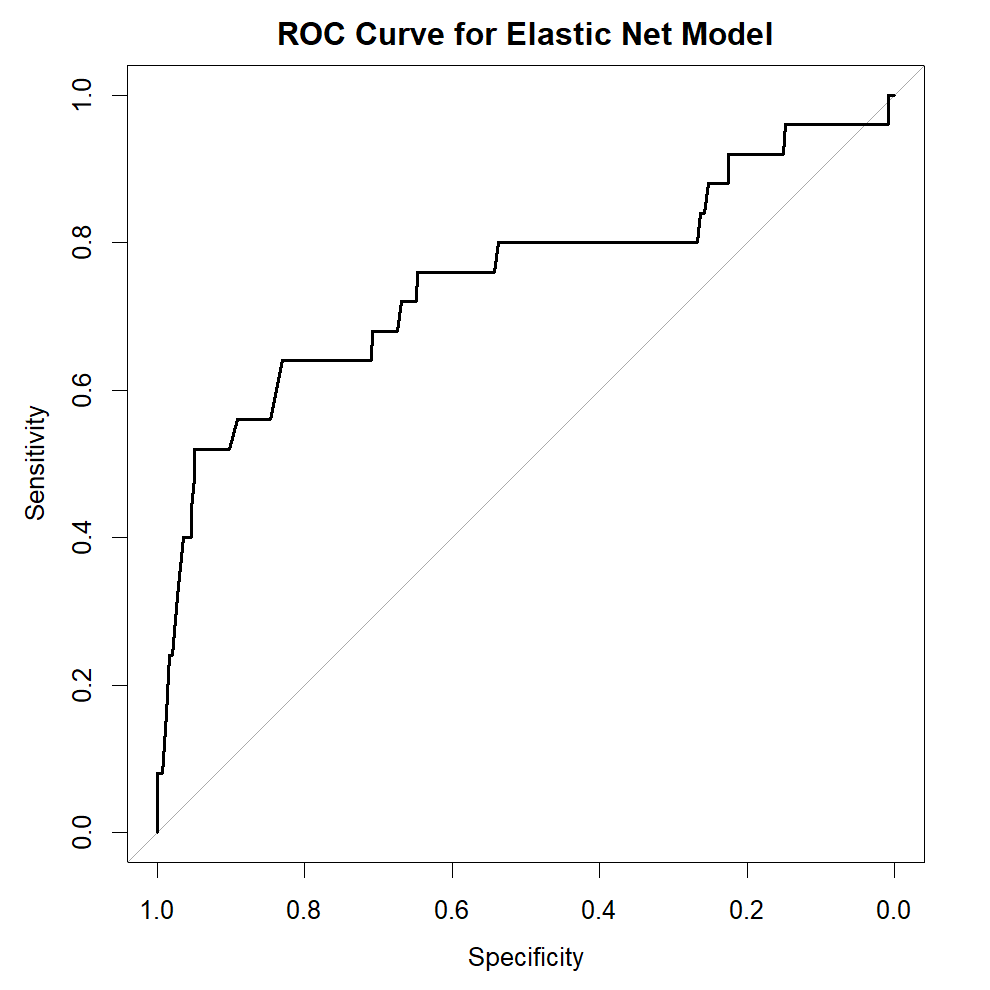


**Supplementary 6. Elastic Net predictions of hospitalization risk by age in RSV+ children, with and without co-infection**

**
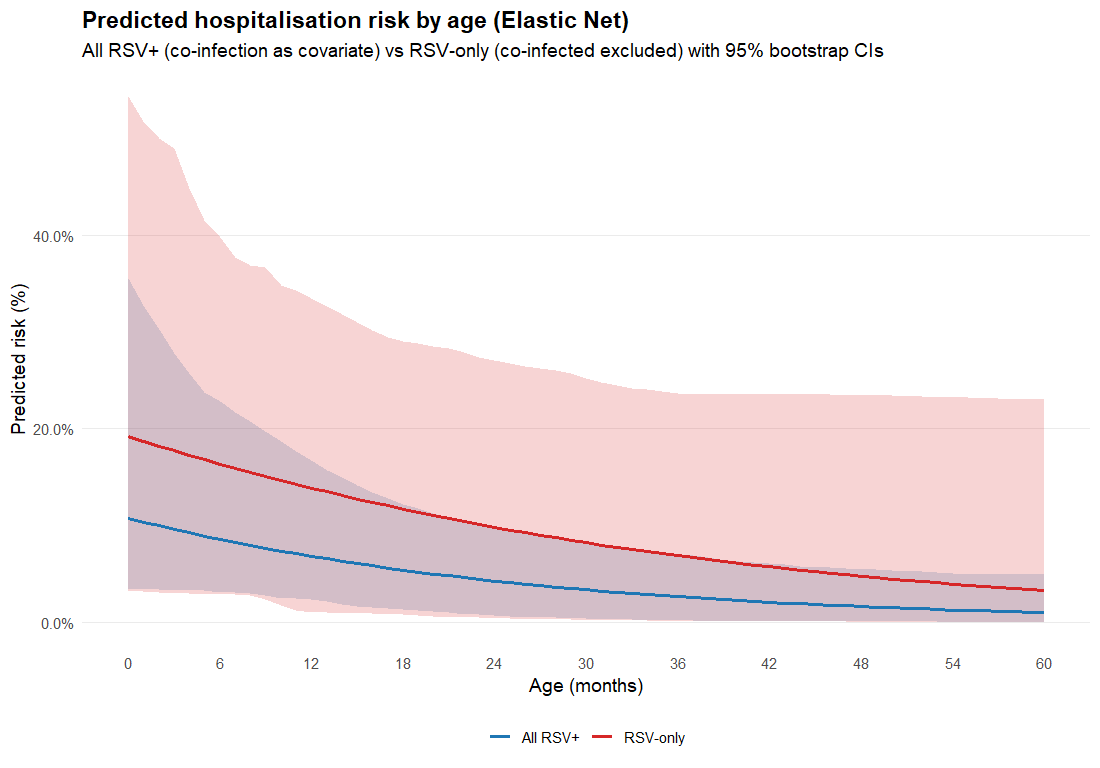
**

Predicted risk of hospitalization by age among RSV-positive children, estimated using Elastic Net models. Curves represent predicted risk for two groups: All RSV+ (all RSV-positive cases, including co-infection as a covariate) and RSV-only (restricted to RSV-positive cases without documented co-infection). Shaded areas indicate 95% bootstrap confidence intervals (CI).

**Supplementary 7. Sensitivity analysis Hospitalization Risk Prediction Model stratified by age for RSV+ only children (no co-infections)**

| **alpha** | **lambda** | **AUC** |
| --- | --- | --- |
| corresponding to RIDGE regression 0·0 | 0·0605 | 0·808 |
| 0·1 | 0·0250 | 0·819 |
| 0·2 | 0·0317 | 0·823 |
| 0·3 | 0·0211 | 0·823 |
| 0·4 | 0·0174 | 0·823 |
| 0·5 | 0·0202 | 0·823 |
| 0·6 | 0·0153 | 0·824 |
| 0·7 | 0·0158 | 0·823 |
| 0·8 | 0·0139 | 0·824 |
| 0·9 | 0·0112 | 0·825 |
| corresponding to LASSO regression 1·0 | 0·0101 | 0·827 |
| **Final model results** |  |  |
| **Selected model** |  |  |
| alpha | 1·0 |  |
| lambda | 0·01 |  |
| AUC | 0.827 |  |
|  |  |  |
| **Model parameters (beta)** |  |  |
| age | -0·0327 |  |
| sex (male) | · |  |
| prematurity (fullterm) | · |  |
| season (2021-2022) | - |  |
| season (2022-2023) | -1·61 |  |
| season (2023-2024) | 0.283 |  |
| other pathogens | - |  |
|  | **Predicted Hospitalization Risk** | **95% CI bootstrapped** |
| **Age (months)** |  |  |
| 0 | 10·7% | 3·4 - 35·6 % |
| 3 | 9·6% | 3·3- 27·8 % |
| 6 | 8·6% | 3·1 - 22·8 % |
| 12 | 6·8% | 2·3 - 16·7 % |
| 24 | 4·2% | 0·6 - 9·8 % |

**Supplementary 8. Calibration of Elastic Net Regression Hospitalization Risk Prediction Model**

We assessed calibration using 10-fold out-of-fold (OOF) predictions from the elastic-net logistic model described in Supplementary 5. Folds were grouped by recruiting site. Within each training fold, we used internal 5-fold cross-validation to choose the elastic-net tuning parameters by minimizing binomial deviance, then generated OOF probabilities for the held-out fold. From these OOF predictions, we report the AUC, Brier score, calibration in the large, and the calibration slope, along with 95% Wald confidence intervals.

**Results:** AUC 0.645; Brier 0.042; calibration intercept −0.002 (95% CI −0.407, 0.402); calibration slope 0.498 (95% CI −0.135, 1.131).

**Figure S8. Calibration of the hospitalization risk model (elastic net).**

**
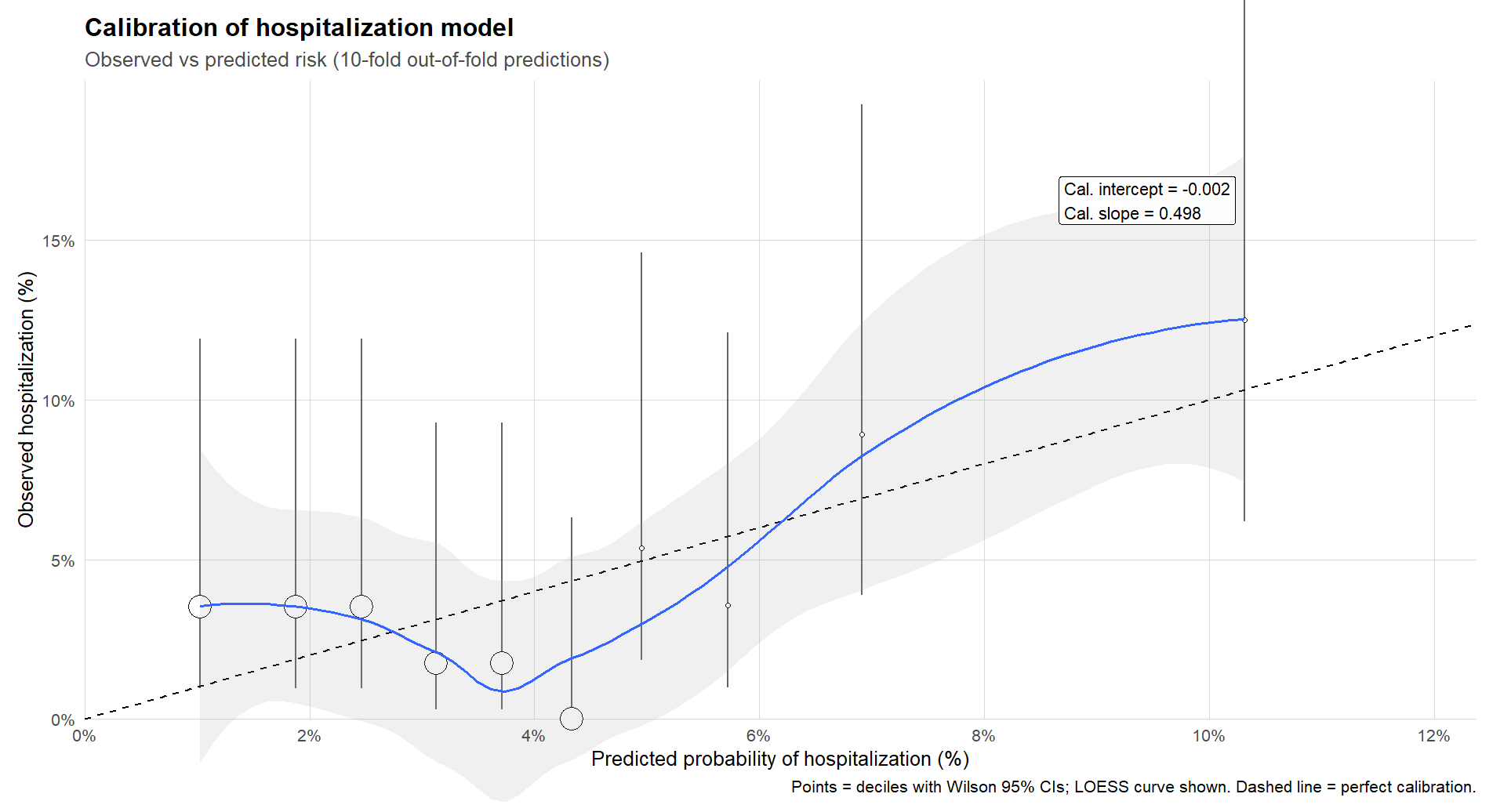
**

Observed versus predicted probability of hospitalization using 10-fold out-of-fold (OOF) predictions. Points represent deciles of predicted risk with Wilson 95% CIs; the solid curve is a LOESS smoother, and the dashed 45° line indicates perfect calibration. Axes are shown as percentages and zoomed to the observed range of predictions for readability.

**Supplementary 9. Discrimination by Centre of Elastic Net Regression Hospitalization Risk Prediction Model**

We quantified heterogeneity in discrimination across centres using a leave-one-site-out (LOSO) evaluation. For each centre *s*, the elastic-net logistic model was trained on all other centres and used to predict outcomes in *s*. Within the held-out centre, we computed the AUC and 95% CI . Centres with no events or no non-events were labeled not estimable.

**Results:** Under LOSO, site-level AUCs (95% CI; events in parentheses) were: Lazio 0.415 (0.074–0.755; 5), Liguria 0.622 (0.362–0.883; 7), Lombardia 0.590 (0.000–1.000; 2), Puglia 0.695 (0.601–0.788; 11); Toscana not estimable (0 events). The weighted mean AUC across centres was 0.592.

**Figure S9. Centre-specific discrimination for the elastic-net hospitalization risk model**


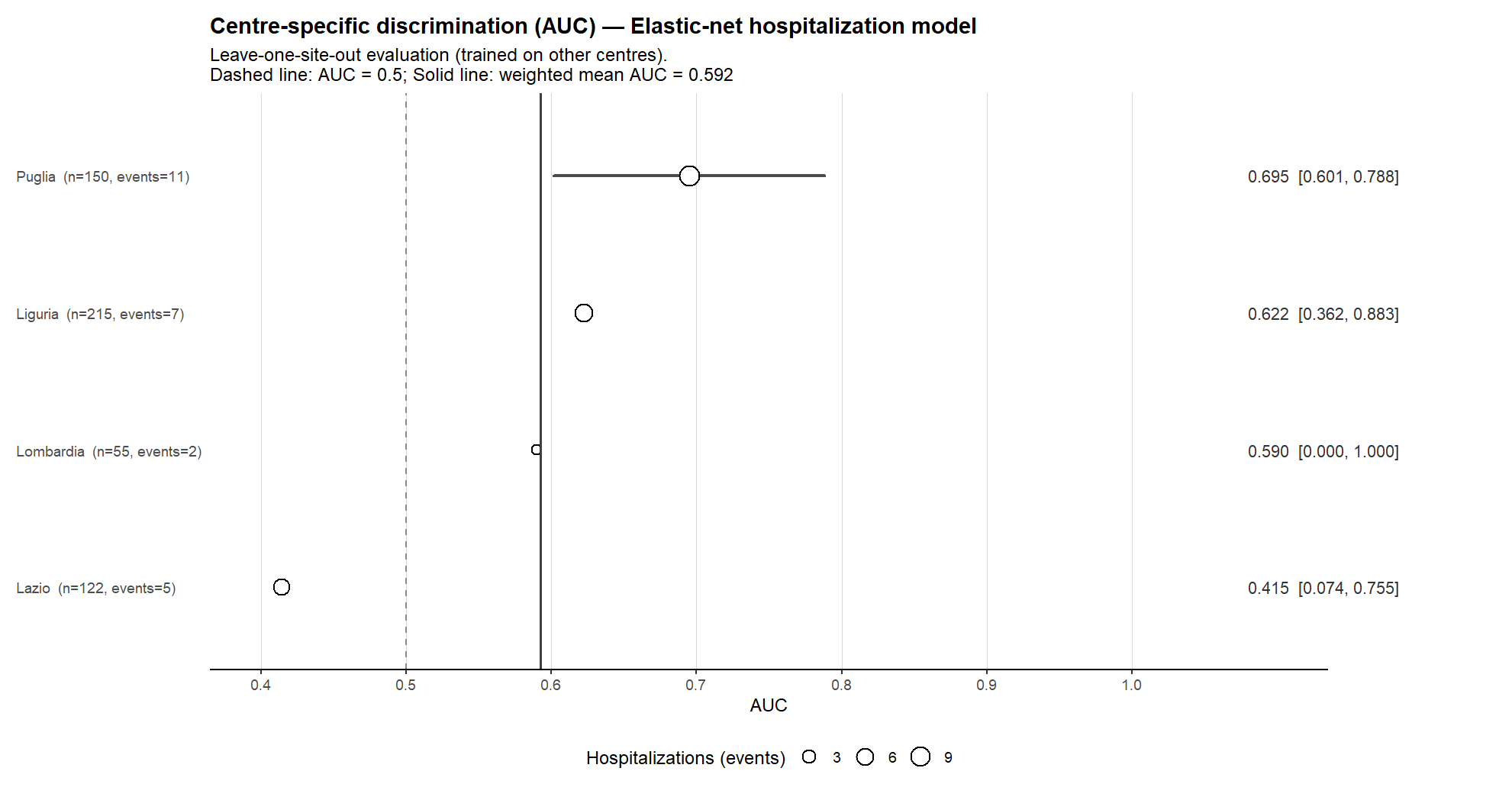


Forest plot of site-level AUCs with 95% confidence intervals under leave-one-site-out (LOSO) evaluation. Points (sized by number of hospitalizations/events) mark the AUC; horizontal lines show 95% CIs. The dashed vertical line indicates AUC = 0.5 (no discrimination), and the solid vertical line indicates the weighted mean AUC across centres. Centres with no events were not estimable and are listed separately.
