# Supplementary material for: Clinical presentation and hospitalisation risk of RSV in primary care among children younger than 5 years in Italy in four seasons between 2019 and 2023: a multicentre prospective cohort study
Source: Lancet Reg Health Eur. 2026 Mar 24;65:101652. doi: 10.1016/j.lanepe.2026.101652 (PMC13049889; doi:10.1016/j.lanepe.2026.101652)
Supplement: Translated Abstract [file mmc2.docx]

Introduzione

Il virus respiratorio sinciziale (VRS) rappresenta una causa significativa di mortalità infantile a livello globale. Sebbene i casi severi siano più comuni fra neonati e bambini con comorbilità, anche i casi meno gravi possono esitare in problemi respiratori cronici. L’epidemiologia di VRS nel contesto ospedaliero è ben caratterizzata, mentre la presentazione clinica e il rischio di ospedalizzazione nel contesto territoriale dell’assistenza primaria restano poco esplorati.

Metodi

È stato condotto uno studio di coorte multicentrico in diverse regioni italiane durante quattro stagioni influenzali (dal 2019-2020 al 2023-2024), con l’esclusione del 2020-2021. Attraverso un network di pediatri di libera scelta, sono stati arruolati bambini di età inferiore a 5 anni che soddisfacevano la definizione dell'OMS di sorveglianza comunitaria delle infezioni respiratorie acute (ARI). Durante la visita di arruolamento è stata raccolta un’anamesi e sono stati eseguiti tamponi nasofaringei, successivamente analizzati con RT-PCR; in seguito, i bambini risultati positivi per VRS sono stati seguiti telefonicamente con follow-up a 14 e 30 giorni per la valutazione delle condizioni cliniche.

Risultati

Tra i 1410 bambini arruolati, il 40·2% è risultato positivo per VRS all’analisi di laboratorio. La durata media della malattia da VRS è stata di 15·2 giorni (SD 8·8 giorni), con sintomi ancora presenti a 14 giorni nel 40·9% dei casi e a 30 giorni nel 15·4% dei casi. Il 4·4% (25/566) dei casi VRS positivi ha richiesto l’ospedalizzazione, con una durata mediana di 5 giorni (IQR: 4–7 giorni). L’età all’infezione è stata identificata come il principale predittore dell’ospedalizzazione (con un tasso di ospedalizzazione osservato del 16·2% nei bambini al di sotto dei sei mesi di età) e con un rischio stimato alla nascita del 12·5% (95% CI: 3·4–33·1%). La presentazione clinica con febbre non si associa in modo significativo con l’infezione da VRS (p=0·084).

Conclusioni

Lo studio evidenzia l’elevato rischio di ospedalizzazione dovuto alle infezioni da VRS nei bambini assistiti a livello territoriale, fornendo stime del rischio specifico per età basate su un modello predittivo, fondamentali per le valutazioni in ambito di Health Technology Assessment (HTA) relative alle strategie preventive.

I risultati supportano l’ampliamento delle azioni preventive nei bambini oltre la soglia dei 24 mesi di età, evidenziando la persistenza del rischio di ospedalizzazione anche nei soggetti di età compresa tra 2 e 5 anni. Inoltre, sottolineiamo l’importanza di adottare una definizione di caso che non richieda necessariamente la presenza di febbre, al fine di aumentare la sensibilità della sorveglianza VRS e migliorare l’accuratezza delle stime della malattia.

Finanziamento

Lo studio ha ricevuto supporto finanziario non condizionante da Sanofi Pasteur e Astrazeneca tramite NIVEL.
