# Supplementary material for: Clinical presentation and hospitalisation risk of RSV in primary care among children younger than 5 years in Italy in four seasons between 2019 and 2023: a multicentre prospective cohort study
Source: Lancet Reg Health Eur. 2026 Mar 24;65:101652. doi: 10.1016/j.lanepe.2026.101652 (PMC13049889; doi:10.1016/j.lanepe.2026.101652)
Supplement: Collab Authors [file mmc3.docx]

RSV ComNet Ita Network

| Nicolò | Di Gaddo |
| --- | --- |
| Michela | Scarpaci |
| Tommaso | Cosci |
| Luigi | De Angelis |
| Melissa | Torrisi |
| Leonardo | Bonaldo |
| Guglielmo | Arzilli |
| Gianluca | Paparatto |
| Virginia | Casigliani |
| Andrea Davide | Porretta |
| Federica | Chiovelli |
| Yasmine | Ferchichi |
| Maria | Sidoti |
| Anna Lisa | Capria |
| Valerio | Corsini |
| Flavia | Favilli |
| Paola | Mazzetti |
| Loredana | Costabile |
| Rossella | Cannavò |
| Micaela | Foco |
| Valentina | Grimaldi |
| Immacolata | Labella |
| Roberta | Lanni |
| Donatella | Morano |
| Paolo | Nardini |
| Fabrizio | Piperno |
| Claudia | Pontesilli |
| Innocenza | Rafele |
| Laura | Reali |
| Teresa | Rongai |
| Michele | Valente |
| Laura | Venuti |
| Piero Luigi | Lai |
| Carola | Minet |
| Carlo Simone | Trombetta |
| Chiara | Amoruso |
| Alessia | De Marzo |
| Miriana | Girardi |
| Lucia | Servedio |
| Lucia | Peccarisi |
| Giovanni | Capaldi |
| Daniela | Damiani |
| Vincenzo | Frappampina |
| Stefania | Frau |
| Nunzio | Guglielmi |
| Giuseppe | Ragnatela |
| Monica | Pepe |
| Cristoforo | Vania |
| Cristina | Galli |
| Sandro | Binda |
| Paola | Guidotti |
| Arianna | Passoni |
| Patrizia | Rogari |
| Luigi | Greco |
